# Prevalence and consequences of musculoskeletal symptoms in symphony orchestra musicians vary by gender: a cross-sectional study

**DOI:** 10.1186/1471-2474-12-223

**Published:** 2011-10-07

**Authors:** Helene M Paarup, Jesper Baelum, Jonas W Holm, Claus Manniche, Niels Wedderkopp

**Affiliations:** 1 Research Unit of Occupational and Environmental Health, Institute of Clinical Research, Faculty of Health Sciences, University of Southern Denmark, Odense, Denmark; 2 Department of Occupational and Environmental Medicine, Odense University Hospital, Odense, Denmark; 3 Department of Occupational Medicine, Koege Hospital, Koege, Denmark; 4 The Research Department, Spine Center of Southern Denmark, Hospital Lillebaelt, Middelfart, Denmark; 5 Institute of Regional Health Services, Faculty of Health Sciences, University of Southern Denmark, Odense, Denmark

## Abstract

**Background:**

Musculoskeletal symptoms are common in the neck, back, and upper limbs amongst musicians. Playing-related musculoskeletal disorders have been found to range from 32% to 87% with a tendency for female musicians to have more problems than males. Studies of musculoskeletal problems in instrumentalists have generally involved pre-professional musicians or populations comprising musicians of different levels. The objective of this study was therefore to investigate the prevalence, duration and consequences of musculoskeletal symptoms in professional symphony orchestra musicians.

**Methods:**

A cross-sectional questionnaire study. The study population comprised of 441 musicians from six Danish symphony orchestras; 342 (78%) completed the questionnaire.

**Results:**

During the last year 97% of the women and 83% of the men experienced symptoms in at least one of nine anatomic regions (neck, upper and lower back, shoulders, elbows, and hands and wrists). 86% of the women and 67% of the men experienced symptoms for more than seven days, while 63% of the women and 49% of the men had symptoms for more than 30 days. Woodwind players had a lower risk for musculoskeletal symptoms and a lower risk for the consequences. Among consequences were changed way of playing, reported by 73% of the musicians, difficulty in daily activities at home, reported by 55%, and difficulty in sleeping, reported by 49%. Their health behaviour included taking paracetamol as the most used analgesic, while physiotherapists and general practitioners were reported as the most consulted health care professionals concerning musculoskeletal problems.

Results regarding symptoms in six anatomic regions were compared to results for a sample of the general Danish workforce. Symptoms were more frequent in musicians and lasted longer than in the general workforce. This applied to both genders.

**Conclusions:**

Within the last year most symphony orchestra musicians experienced musculoskeletal symptoms in the neck, back or upper extremities. The symptoms impacted on their level of function in and outside work and were reflected in their health behaviour. Generally women had a higher risk than men and woodwind players a lower risk than other instrumentalists. Finally, symptoms were more frequent and lasted longer in the musicians than in the general workforce.

## Background

Across countries and continents, many professional classical musicians are employed in symphony orchestras that are generally comparable in instrumentation and in the hierarchical organization of their different instrument groups [1,2]. The ergonomic exposure from each kind of instrument, the need to practice for many hours to remain at the elite level, and the importance of each musician performing well during a concert, is also comparable worldwide. Despite the international relevance of this occupational group, little research has been done to understand more about its musculoskeletal problems and to investigate to what extent the occurrence of musculoskeletal disorders has an influence on the symphony orchestra musicians' level of function.

In the scientifically biomedical literature, professional symphony orchestra musicians are poorly described, as most studies of musculoskeletal problems in musicians have been done with populations involving pre-professional musicians, or mixed populations with different types of musicians, and thereby mixed exposures [3,4]. The research carried out on musculoskeletal symptoms has used different measures and the musician-specific term "playing-related musculoskeletal disorders" is not uniquely defined [3-5]. A possible limitation inherent in playing-related measures is the exclusion of musculoskeletal symptoms acquired for other reasons than from music playing, symptoms which can still impact on music playing, and another limitation is the focusing on the impact on playing neglecting possible consequences outside playing-situations. Nevertheless the literature indicates that musculoskeletal symptoms in the neck, back, and upper extremities are common in musicians of different levels. A gender difference has been reported as well; there is a tendency for female musicians to have more problems than male musicians [6-8]. Moreover the prevalence of playing-related musculoskeletal disorders in musicians has been found to range from 32% to 87% [3,9].

Symphony orchestra musicians are often music conservatorium graduates and have practiced for hours every day to reach the elite level. This level can be achieved only through effort and discipline and with the will to improve their techniques all the time [2,10]. They usually play one main instrument throughout their entire career and play about 1,300 hours a year [11]. Symphony orchestra musicians are trained in a competitive environment where a high level of proficiency has to be maintained [10]. Vacant positions in the orchestras are normally filled by audition winners, as employment is generally preceded by an audition. Consequently, a high degree of healthy worker selection could be expected [12]. Moreover, the evolution of the classical musical instruments has been based on tradition and sound and little consideration was given to designing an up-to-date work tool to be played for hours every day. The instruments are played with little scope for variation in neck, trunk, and upper extremity positions and consequently, most musicians have ergonomically monotonous work strain, often including static positions of the neck, static and/or dynamic shoulder load, repetitive or static elbow work, wrist flexion, and dynamic finger movements [9,13-15]. Hence, symphony orchestra musicians are likely to acquire work-related musculoskeletal problems, as static and repetitive work characteristics in many occupational settings have been associated with musculoskeletal disorders in the neck, back and upper extremities [16-20].

The aim of this study was to investigate the prevalence and consequences of perceived musculoskeletal symptoms in the highly selected occupational group of symphony orchestra musicians.

## Methods

### Design

This research involved a cross-sectional questionnaire study of professional symphony orchestra musicians in Denmark.

### Population

All seven professional symphony orchestras in Denmark were asked to participate in the study. They were included orchestra by orchestra to ensure that all employed musicians could receive information and participate in the survey at their workplace. One orchestra was not able to participate due to administrative reasons and was not included. In the remaining orchestras, four musicians on long-term leave could not be contacted and were not included. Nobody was excluded from the study. The study population was thus 441 musicians from the six participating symphony orchestras, with 342 (78%) answering the questionnaire, in total 208 men and 134 women. The respondents, as well as the study population, were comprised of 61% men and 39% women.

### Reference Population

Results from the symphony orchestra musicians were compared to results from The Danish Working Environment Cohort, a study performed by The National Research Centre for Work Environment and Health, Denmark, who provided the data concerning the cohort for reference use in the current study. The Danish Working Environment Cohort Study is a sample-based, multi-topic survey of work environment and health among the Danish workforce [21]. The sample was a representative sample from the Danish workforce, where the distribution of gender, age, labour market attachment, and geography was the same as for the general population in Denmark [22]. The reference data comprising 2,731 men and 2,705 women were collected through interviews in the Year 2000 [23].

### Setting

Data were collected from January 2007 to June 2008. All contacts with the participants took place at the musicians' workplace. The orchestras were visited one at a time. An introductory meeting was held, after which the questionnaires were distributed to each musician. The questionnaires were completed during work-time and collected less than two hours later. The few musicians who could not attend the introductory meeting received an information letter and a questionnaire, both delivered to their private mailbox at work.

### Variables

Gender, age, orchestra of employment and instrument information were obtained for all participants in the study population as baseline data from employment lists in the participating orchestras.

In accordance with the aim of study, the main outcomes were information about musculoskeletal symptoms and consequences of the symptoms. The musculoskeletal symptoms were measured as 1) period prevalences: the 7 days' and 12 months' prevalence, and 2) symptoms duration: symptoms for more than 7 days and for more than 30 days. The consequences were measured as 1) impact on level of function on work: impaired or changed way of playing, 2) impact on level of function outside work: difficulty in daily activities at home, difficulty in leisure time activities, or difficulty in sleep, and 3) behavioural consequences: pausing from practicing alone, pausing from rehearsals, not playing at concerts, taking sick-leave, using painkillers, or consulting health care providers.

Work-related questions included playing exposure, music education and current employment.

### Questionnaire construction

The questionnaire was constructed on the basis of interviews with classically trained musicians. Questions regarding musculoskeletal symptoms were adapted from the Nordic Musculoskeletal Questionnaire [24-26]. The symptoms were measured as presence of trouble (ache, pain, or discomfort) within the last 7 days and the last 12 months in the different anatomic regions. Furthermore questions were asked about the number of days with symptoms within the last 12 months. The questions were for each anatomic region supplemented with newly developed questions about the impact of the musculoskeletal symptoms. The questions regarding the level of function in and outside work were inspired by the high performance sport/music module in the questionnaire entitled Disabilities of the Arm, Shoulder, and Hand (DASH) [27]. As in the DASH questionnaire four questions were asked to assess if the symptoms had led to difficulties in playing, but the time span of interest was extended to 12 months. In a similar way of asking followed questions about whether the symptoms had led to difficulties in daily activities at home, in leisure time activities, or in sleeping. Questions about how the musicians coped with the musculoskeletal symptoms included sick-leave, if they had had to pause from practicing or performing music, and their utilisation of health care providers and analgesics due to musculoskeletal symptoms was also investigated. Types of analgesics were specified in a separate questionnaire item regarding medicines consumption.

### Validity

The face validity of the draft questionnaire was tested with help from music conservatory students from the Carl Nielsen Academy of Music, Odense, Denmark, who filled in the questionnaire and commented on the comprehensibility of the questionnaire, any disambiguity of questions, and the relevance of the topics. The face validity of the final questionnaire was tested by an expert panel consisting of professionals from the Danish music industry and from the musicians' union.

### Statistical methods

In the descriptive analysis, all crude prevalence calculations were based on the total group of respondents by gender, the denominator always being 208 for men and 134 for women. The used prevalence calculations for the reference group were likewise based on the total group by gender. Test for equality of proportions was used for calculating the level of significance of different results in comparable groups, e.g. between genders or between the musicians and the general workforce. Associations between the binary main health outcomes and instrument groups were estimated using logistic regression with robust standard error adjusting for gender, age, number of playing years on the main instrument, and using orchestra of employment as the cluster variable. The high string group accounted for the largest instrument group and was chosen as reference in the analysis. Likewise associations between health outcomes and gender were estimated using robust logistic regression adjusted for age, instrument group, number of playing years on the main instrument, and with orchestra of employment as the cluster option; being the largest group, men were chosen as reference. Exactly the same method was used to estimate associations between the consequences of the musculoskeletal symptoms and gender and instrument groups. Prevalence odds ratios were calculated with 95% confidence intervals. The level of significance was defined as p < 0.05. Statistical analysis was performed using Stata 10.0 (StataCorp LP, College Station, Texas, US).

### Ethics

Approval for this study was obtained from the Danish Data Protection Agency (journal no. 2006-41-7194) and the Research Ethics Committee (project ID VF-20060086). Written information about the study was provided to all participants and meetings were held to provide oral information. Participation was voluntary.

## Results

### Music education and employment

Most of the musicians have played music, and often their main instrument, since childhood, see Table 1. Of the men, 69% had acquired a music academy master degree compared with 80% of the women. In all age groups of the symphony orchestra, more musicians were permanently than temporarily employed, and 90.4% of the males and 88.8% of the women had permanent employment. Of those temporarily employed, 71% were younger than 40 years of age.

**Table 1:** Time measures and instrument groups.

	Men (N = 208)	Women (N = 134)
	**Median (95% CI)**	**Median (95% CI)**

Age on time of survey, years	48 (46-50)	39 (37-43)

	**Median (95% CI)**	**Median (95% CI)**

***Exposure: time measures***		
Age for starting playing music, years	7 (7 - 8)	6 (6 - 7)
Age for start with main instrument, years	10 (10 - 11)	9 (9 - 10)
Duration of current employment, years	18.3 (15.9 - 20.5)	11.7 (10.0 - 15.0)
Number of playing days/week	6 (6 - 7)	7 (6 - 7)
Total working hours/week incl. secondary job	32.0 (31.0 - 34.0)	32.0 (31.0 - 34.0)
Total playing hours/week	31.0 (30.0 - 32.0)	32.0 (30.0 - 33.0)
-hereof practicing alone	7.0 (6.6 - 9.0)	9.0 (7.0 - 10.0)
-hereof practicing/rehearsing with colleagues	18.0 (16.0 - 20.0)	18.0 (16.0 - 20.0)
-playing concert/performing	4.0 (4.0 - 4.0)	4.0 (4.0 - 4.0)

	**N (Percent)**	**N (Percent)**

***Participants by instrument groups***		
High strings (violin, viola)	63 (30.3%)	86 (64.2%)
Low strings (cello, double bass)	43 (20.7%)	16 (11.9%)
Woodwinds (flute, oboe, clarinet, bassoon)	41 (19.7%)	21 (15.7%)
Brass (horn, trumpet, trombone, tuba)	46 (22.1%)	7 (5.2%)
Others (percussion, tympani, harp, keyboards)	15 (7.2%)	4 (3.0%)
All instrument groups	208 (100.0%)	134 (100%)

Women were significantly younger than men and had fewer years in the current employment. The time exposure measures were in general very similar for both genders except that women practiced more alone than men. The major part of the women played high strings, the only instrument group dominated by women.

### Prevalence and duration of symptoms

**Figure 1:**
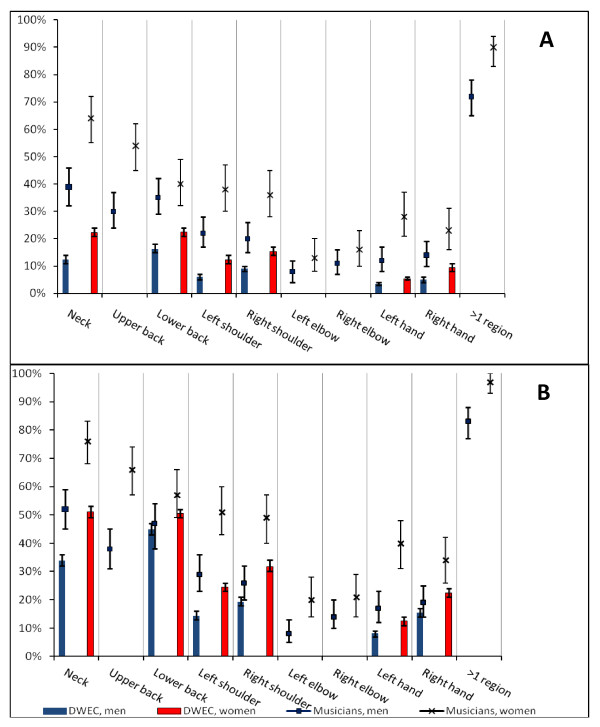
**Prevalence of symptoms in symphony orchestra musicians and comparison with The Danish Work Environment Cohort**. A) Prevalence of musculoskeletal symptoms within the previous 7 days. B) Prevalence of musculoskeletal symptoms within the previous 12 months. No data were available on upper back, elbows, and > 1 region for DWEC (The Danish Work Environment Cohort). The error bars show the 95% confidence interval. DWEC: 2,731 men and 2,705 women. Musicians: 208 men and 134 women.

**Figure 2:**
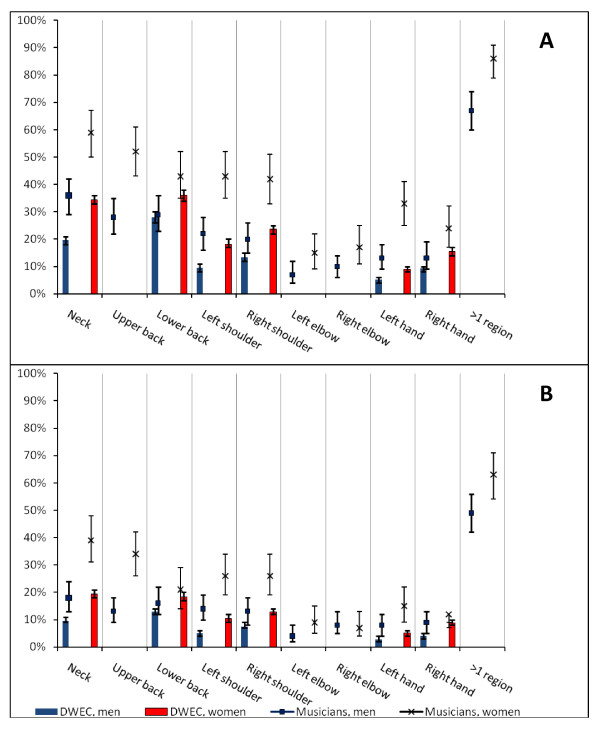
**Days of symptoms in symphony orchestra musicians and comparison with The Danish Work Environment Cohort**. A) Prevalence of musculoskeletal symptoms for more than 7 days within the previous 12 months. B) Prevalence of musculoskeletal symptoms for more than 30 days within the previous 12 months. No data were available on upper back, elbows, and > 1 region for DWEC (The Danish Work Environment Cohort). The error bars show the 95% confidence interval. DWEC: 2,731 men and 2,705 women. Musicians: 208 men and 134 women.

The prevalence of reported musculoskeletal symptoms in nine anatomic regions are shown by gender in Figure 1 and duration of symptoms in Figure 2, the error bars illustrating the 95% confidence interval. For all regions, the female symphony orchestra musicians reported more symptoms, and significantly more than men for the neck and upper back, left and right shoulder, left hand and wrist. Symptoms in at least one of the regions within the last year were reported by 97% of the women and 83% of the men. Seven days or more of symptoms were experienced by 86% of the women and 67% of the men, and 63% of the women and 49% of the men had the problems for more than 30 days within the last year.

Prevalence odds ratios with 95% confidence intervals for the different anatomic regions are listed by gender in Table 2. Symptoms for most anatomic regions were significantly associated with being of female gender, and for symptoms in at least one of nine regions, women had a statistically significantly higher odds ratio of 6.5 (p = 0.000; CI 2.3-18.2) in the 12 months' prevalence, and of 3.0 (p = 0.000; CI 1.9-4.5) in the one-week prevalence. For symptoms of more than seven days within the last year in at least one region the women had an odds ratio of 2.7 (p = 0.000; CI 2.3-3.3), and for symptoms more than 30 days within the last 12 months, women had an odds ratio of 2.2 (p = 0.015; CI 1.2-4.1) compared to men. All odds ratios were 1.0 for men, as they were the reference.

**Table 2:** Prevalence odds ratios for musculoskeletal symptoms by gender.

		Symptoms within the previous 12 months	Symptoms within the previous 7 days	Symptoms > 7 days within the previous 12 months	Symptoms > 30 days within the previous 12 months
	**Men** **(N = 208)**	**Women** **(N = 134)**	**Women** **(N = 134)**	**Women** **(N = 134)**	**Women** **(N = 134)**

	**OR**	**OR (95% CI)**	**OR (95% CI)**	**OR (95% CI)**	**OR (95% CI)**

Neck	1.0	**2.9 **(1.9 - 4.6)	**2.8 **(2.0 - 4.1)	**2.4 **(1.6 - 3.7)	**3.6 **(1.9 - 7.1)
Upper back	1.0	**2.8 **(2.1 - 3.8)	**2.6 **(2.0 - 3.3)	**2.6 **(1.9 - 3.5)	**4.4 **(3.6 - 5.5)
Lower back	1.0	1.3 (0.8 - 2.4)	1.1 (0.7 - 2.0)	1.8 (0.9 - 3.5)	**1.5 **(0.8 - 2.9)
Left shoulder	1.0	**2.4 **(1.6 - 3.7)	1.6 (0.8 - 3.4)	**2.7 **(1.4 - 5.5)	**2.6 **(1.3 - 5.0)
Right shoulder	1.0	**3.2 **(1.8 - 5.6)	**2.6 **(1.6 - 4.4)	**3.5 **(1.9 - 6.5)	**3.1 **(1.4 - 7.2)
Left elbow	1.0	**3.5 **(1.2 - 10.1)	2.2 (0.6 - 8.3)	2.7 (0.9 - 8.3)	**2.7 **(1.0 - 6.9)
Right elbow	1.0	1.7 (0.8 - 3.6)	**1.8 **(1.0 - 3.2)	**1.9 **(1.0 - 3.4)	0.8 (0.4 - 1.9)
Left hand & wrist	1.0	**3.3 **(1.6 - 7.2)	**3.7 **(2.3 - 6.0)	**3.8 **(1.6 - 8.8)	**3.4 **(1.3 - 8.8)
Right hand & wrist	1.0	**2.1 **(1.5 - 3.0)	**2.1 **(1.3 - 3.3)	**2.5 **(1.4 - 4.4)	**2.2 **(1.1 - 4.4)
≥1 anatomic region	1.0	**6.5 **(2.3 - 18.2)	**3.0 **(1.9 - 4.5)	**2.7 **(2.3 - 3.3)	**2.2 **(1.2 - 4.1)

Association between musculoskeletal symptoms in different anatomic regions and gender expressed as prevalence odds ratios. The odds ratios are results of logistic regression. All analyses were adjusted for age, instrument group, number of playing years on main instrument, and had orchestra of employment as cluster variable. Prevalence odds ratios with P-value < 0.05 are marked with bold. For all outcomes the odds ratio was 1.0 for men, men being the reference group.

The prevalence odds ratios of symptoms were also calculated by instrument group, see Table 3. For the majority of instrument groups, there was barely any significant association between the symptoms in the different anatomic regions and the main instrument groups. However, for symptoms within the last 7 days as well as symptoms within the last 12 months, the woodwind section had a significantly lower odds ratio for symptoms in the neck, left shoulder, and symptoms in at least one region compared to the group of high strings. For symptoms within the last 12 months the woodwinds also had a significantly lower odds ratio regarding the lower back. For brass players the odds ratio for experienced symptoms in the right hand and wrist within the last 12 month was found statistically lower compared to the high strings.

**Table 3:** Prevalence odds ratios for musculoskeletal symptoms by instrument groups.

	High strings (N = 149)	Low strings (N = 59)	Woodwinds (N = 62)	Brass players (N = 53)	Others (N = 19)
	**OR**	**OR (95% CI)**	**OR (95% CI)**	**OR (95% CI)**	**OR (95% CI)**

***Symptoms within the previous 12 months in the***					
Neck	1.0	1.0 (0.6 - 1.6)	**0.5 **(0.3 - 0.7)	0.8 (0.3 - 2.1)	0.6 (0.3 -1.6)
Upper back	1.0	1.4 (0.6 -3.0)	1.0 (0.5 - 2.0)	0.9 (0.4 - 1.9)	1.5 (0.8 - 2.9)
Lower back	1.0	0.7 (0.4 - 1.5)	**0.5 **(0.3 - 0.8)	0.8 (0.3 - 2.2)	0.8 (0.2 - 3.2)
Left shoulder	1.0	0.6 (0.3 - 1.1)	**0.5 **(0.3 - 0.8)	1.2 (0.6 - 2.4)	**0.3 **(0.1 - 0.8)
Right shoulder	1.0	1.7 (0.7 - 3.9)	0.8 (0.3 - 2.1)	1.3 (0.6 - 2.7)	0.8 (0.1 - 5.2)
Left elbow	1.0	1.5 (0.6 - 3.9)	0.4 (0.1 - 1.9)	1.7 (0.9 -3.4)	**4.7 **(1.2 - 18.4)
Right elbow	1.0	1.1 (0.5 - 2.5)	1.0 (0.4 - 2.6)	0.6 (0.2 - 2.1)	1.2 (0.4 - 3.4)
Left hand & wrist	1.0	1.3 (0.7 - 2.6)	0.5 (0.2 - 1.2)	0.8 (0.4 - 1.8)	1.1 (0.2 - 6.7)
Right hand & wrist	1.0	1.8 (0.8 - 3.9)	1.2 (0.5 - 2.7)	**0.4 **(0.2 - 0.8)	1.8 (0.4 - 7.5)
≥ 1 anatomic region	1.0	0.9 (0.4 - 2.2)	**0.5 **(0.3 - 0.9)	1.4 (0.4 - 4.4)	0.4 (0.1 - 1.5)
***Symptoms within the previous 7 days in the***					
Neck	1.0	1.4 (0.8 - 2.6)	**0.5 **(0.3 - 0.8)	0.8 (0.3 - 2.1)	0.8 (0.2 - 2.6)
Upper back	1.0	1.3 (0.7 - 2.4)	0.8 (0.4 - 1.5)	0.8 (0.3 - 2.3)	1.7 (0.7 - 3.8)
Lower back	1.0	1.1 (0.5 - 2.4)	0.5 (0.2 - 1.1)	0.9 (0.4 - 2.3)	0.6 (0.1 - 2.7)
Left shoulder	1.0	0.6 (0.3 - 1.5)	**0.5 **(0.3 - 0.9)	0.7 (0.2 - 1.7)	0.4 (0.1 - 1.0)
Right shoulder	1.0	1.6 (0.5 - 4.9)	0.8 (0.3 - 2.3)	1.0 (0.4 - 2.7)	1.2 (0.1 - 11.7)
Left elbow	1.0	1.6 (0.8 - 3.4)	0.4 (0.1 - 1.4)	1.3 (0.4 - 4.6)	4.8 (1.0 - 23.8)
Right elbow	1.0	1.4 (0.6 - 3.6)	1.2 (0.5 - 2.9)	0.8 (0.3 - 2.0)	2.0 (0.6 - 7.0)
Left hand & wrist	1.0	1.4 (0.5 - 3.8)	0.5 (0.2 - 1.3)	1.4 (0.7 - 2.9)	1.7 (0.4 - 7.9)
Right hand & wrist	1.0	2.3 (0.9 - 6.2)	1.9 (0.6 - 5.9)	0.5 (0.2 - 1.3)	3.0 (0.5 - 19.4)
≥ 1 anatomic region	1.0	1.5 (0.8 - 2.8)	**0.5 **(0.3 - 1.0)	0.9 (0.4 - 1.9)	0.5 (0.2 - 1.2)

All analyses were adjusted for gender, age, number of playing years on main instrument, and had orchestra of employment as cluster variable. Prevalence odds ratios with P-value < 0.05 are marked with bold. The instruments of each instrument group are shown in Table 1.

Also regarding the duration of symptoms there was generally little statistically significant association between the instrument played and the reported symptoms, see Table 4. However, low string players had a significantly higher odds ratio for suffering from symptoms for more than 30 days in at least one anatomic region, and specifically in the neck and upper back. The brass players had a higher prevalence odds ratio for more than 30 days of symptoms in the left hand and wrist.

**Table 4:** Prevalence odds ratios for duration of musculoskeletal symptoms within the previous 12 months.

	High strings (N = 149)	Low strings (N = 59)	Woodwinds (N = 62)	Brass players (N = 53)	Others (N = 19)
	**OR**	**OR (95% CI)**	**OR (95% CI)**	**OR (95% CI)**	**OR (95% CI)**

***> 7 days of symptoms in the***					
Neck	1.0	1.3 (0.8 - 2.0)	0.7 (0.4 - 1.1)	0.7 (0.4 - 1.4)	1.0 (0.4 - 2.79
Upper back	1.0	1.4 (0.8 - 2.6)	1.0 (0.5 - 1.9)	0.9 (0.4 - 2.1)	1.6 (0.5 - 4.8)
Lower back	1.0	0.6 (0.3 - 1.0)	0.5 (0.2 - 1.2)	0.9 (0.3 - 2.5)	0.7 (0.3 - 1.7)
Left shoulder	1.0	0.7 (0.3 - 1.6)	**0.5 **(0.3 - 0.9)	1.2 (0.5 - 2.6)	0.4 (0.2 - 1.1)
Right shoulder	1.0	1.6 (0.6 - 3.9)	0.7 (0.3 - 1.6)	1.2 (0.5 - 3.0)	1.1 (0.1 - 7.9)
Left elbow	1.0	1.8 (0.8 - 4.3)	0.4 (0.9 - 1.6)	1.4 (0.4 - 5.3)	3.8 (0.9 - 16.9)
Right elbow	1.0	1.4 (0.6 - 2.9)	0.8 (0.2 - 3.2)	0.3 (0.4 - 3.1)	1.1 (0.3 - 3.7)
Left hand & wrist	1.0	1.2 (0.6 - 2.3)	0.4 (0.1 - 1.0)	0.9 (0.3 - 2.3)	1.1 (0.2 - 5.4)
Right hand & wrist	1.0	**3.2 **(1.4 - 7.3)	1.8 (0.9 - 3.6)	0.3 (0.0 - 2.4)	3.1 (0.5 - 20.4)
≥ 1 anatomic region	1.0	0.9 (0.4 - 2.3)	**0.4 **(0.2 - 0.9)	0.9 (0.3 - 3.3)	0.7 (0.2 - 2.8)
***> 30 days of symptoms in the***					
Neck	1.0	**2.3 **(1.1 - 4.5)	1.1 (0.6 - 1.9)	1.1 (0.4 - 3.4)	1.7 (0.5 - 6.7)
Upper back	1.0	**2.2 **(1.0 - 4.9)	1.4 (0.6 - 3.1)	1.1 (0.7 - 1.6)	2.5 (0.9 -6.9)
Lower back	1.0	1.3 (0.6 - 3.3)	1.0 (0.5 - 1.7)	1.0 (0.3 - 3.9)	1.1 (0.4 - 3.2)
Left shoulder	1.0	1.0 (0.3 - 3.1)	0.9 (0.4 - 1.9)	1.5 (0.6 - 3.7)	1.0 (0.4 - 2.3)
Right shoulder	1.0	2.2 (0.7 - 7.7)	0.8 (0.3 - 2.5)	0.8 (0.2 - 2.9)	0.9 (0.1 - 11.0)
Left elbow	1.0	1.1 (0.6 - 2.2)	0.3 (0.0 - 2.1)	0.9 (0.1 - 7.6)	3.8 (0.7 - 19.4)
Right elbow	1.0	1.2 (0.5 - 2.6)	1.0 (0.3 - 4.0)	0.2 (0.0 - 2.3)	1.8 (0.6 - 5.3)
Left hand & wrist	1.0	1.9 (0.7 - 5.1)	0.6 (0.3 - 1.4)	**2.4 **(1.3 - 4.5)	4.9 (0.7 - 34.9)
Right hand & wrist	1.0	3.0 (0.7 - 12.5)	2.0 (0.8 - 5.0)	0.8 (0.1 - 5.6)	5.5 (0.7 - 43.1)
≥ 1 anatomic region	1.0	**2.3 **(1.1 - 5.0)	0.9 (0.5 - 1.7)	1.1 (0.7 - 1.7)	1.6 (0.4 - 7.0)

All analyses were adjusted for gender, age, number of playing years on main instrument, and had orchestra of employment as cluster variable. Prevalence odds ratios with P-value < 0.05 are marked with bold. The instruments of each instrument group are shown in Table 1.

### Consequences on level of function

Impaired level of function at work, expressed as a changed or impaired way of playing due to symptoms in the neck, back, or upper extremities, was reported by 73%. Regarding impaired level of function outside work due to the musculoskeletal symptoms, 55% reported difficulty in daily activities at home, 53% difficulty in leisure time activities, while 49% had experienced difficulty in sleeping. The prevalence was significantly higher for women regarding changed or impaired playing, difficulty in daily activities at home, and difficulty in sleep.

For impact on leisure time activities, there was no significant gender difference. But according to the adjusted odds ratio, females had a significantly higher risk for experiencing impact on all levels of function due to musculoskeletal symptoms than men, see Table 5.

**Table 5:** Prevalence odd ratios for consequences of musculoskeletal problems by gender.

	Men (N = 208)	Women (N = 134)
	**OR**	**OR (95% CI)**

***Impact on level of function***		
Impaired/changed playing	1.0	**3.3 **(2.4 - 4.5)
Daily activities at home	1.0	**2.8 **(1.9 - 4.2)
Leisure time activities	1.0	**1.9 **(1.6 - 2.3)
Sleep	1.0	**2.7 **(1.9 - 3.9)
***Behavioural consequences***		
Pausing from practicing	1.0	**2.4 **(1.9 - 3.0)
Pausing from rehearsals	1.0	1.6 (0.9 - 2.7)
Omitting playing at concerts	1.0	1.4 (1.0 - 1.9)
Sick-leave	1.0	**1.7 **(1.2 - 2.3)
Use of painkillers	1.0	**2.0 **(1.6 - 2.6)

All analyses were adjusted for age, instrument group, number of playing years on main instrument, and had orchestra of employment as cluster variable. Prevalence odds ratios with P-value < 0.05 are marked with bold.

Regarding the instrument groups the odds ratios for difficulty in playing (impaired or changed way of playing), in daily activities at home, and in doing leisure activities were all statistically significantly lower for the woodwind players compared to the high string players, see Table 6.

**Table 6:** Prevalence odds ratios for consequences of musculoskeletal problems by instrument groups.

	High strings (N = 149)	Low strings (N = 59)	Woodwinds (N = 62)	Brass players (N = 53)	Others (N = 19)
	**OR**	**OR (95% CI)**	**OR (95% CI)**	**OR (95% CI)**	**OR (95% CI)**

***Impact on level of function***					
Impaired/changed playing	1.0	1.0 (0.6 - 1.6)	**0.4 **(0.2 - 0.5)	0.8 (0.3 - 2.2)	**0.3 **(0.2 - 0.5)
Daily activities at home	1.0	**0.7 **(0.5 - 1.0)	**0.4 **(0.3 - 0.7)	0.9 (0.4 - 2.2)	0.7 (0.2 - 2.7)
Leisure time activities	1.0	0.8 (0.6 - 1.1)	**0.3 **(0.2 - 0.8)	1.4 (0.5 - 3.7)	0.9 (0.3 - 3.0)
Sleep	1.0	1.0 (0.6 - 1.7)	0.7 (0.3 - 1.7)	2.0 (0.8 - 4.9)	0.9 (0.3 - 3.0)
***Behavioural consequences***					
Pausing from practicing	1.0	1.0 (0.7 - 1.4)	**0.3 **(0.2 - 0.4)	**0.4 **(0.2 - 0.9)	0.3 (0.1 - 1.3)
Pausing from rehearsals	1.0	0.9 (0.4 - 2.0)	**0.5 **(0.3 - 0.8)	**0.1 **(0.0 - 0.8)	0.2 (0.0 - 2.0)
Omitting playing at concerts	1.0	0.8 (0.3 - 2.1)	**0.3 **(0.1 - 0.5)	**0.2 **(0.1 - 0.5)	0.3 (0.1 - 1.4)
Sick-leave	1.0	0.9 (0.3 - 2.8)	**0.3 **(0.1 - 0.7)	0.3 (0.1 - 1.2)	0.8 (0.2 - 2.9)
Use of painkillers	1.0	0.7 (0.3 - 1.6)	0.5 (0.2 - 1.0)	0.9 (0.5 - 1.7)	0.6 (0.3 - 1.4)

All analyses were adjusted for age, gender, number of playing years on main instrument, and had orchestra of employment as cluster variable. Prevalence odds ratios with P-value < 0.05 are marked with bold. The instruments of each instrument group are shown in Table 1.

### Behavioural consequences

**Figure 3:**
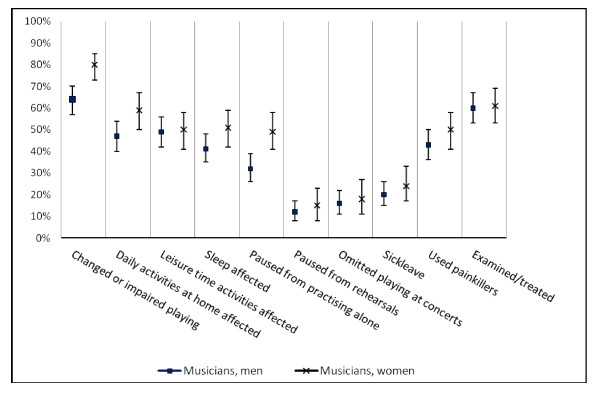
**Consequences of musculoskeletal symptoms in symphony orchestra musicians**. The prevalence of symphony orchestra musicians (208 men, 134 women) who reported having experienced these consequences due to musculoskeletal symptoms in the neck, back or upper extremities within the previous 12 months. The error bars show the 95% confidence interval.

In the previous 12 months, 42% had paused from practicing at home/alone as a consequence of musculoskeletal symptoms. For both genders, this was significantly more frequent than pausing from rehearsals with colleagues, as reported by 16%, and more frequent than not playing at concerts, as reported by 20%. Sick-leave due to neck, back or upper extremity problems was reported by 24%. Use of painkillers due to these symptoms was reported by 49%, neck problems being the most frequently reported cause, and 64% had been examined or treated by a health care professional, see Figure 3.

Looking into the consumption of medicine, whatever the reason, 13% of the women and 12% of the men reported taking mild painkillers such as paracetamol once or more per week, while 44% of the women and 26% of the men reported taking them once or more times per month, making this the most commonly used kind of analgesic. Use of NSAIDs once or more per week was reported by 11% of the women and 8% of the men, while use of NSAIDs once or more per month was reported by 13% of the women and 9% of the men. Regular use of opioids on at least a monthly basis was reported by only 2% of the musicians, sedatives by 6%, and hypnotics by 4%.

The most frequently consulted health care professional for musculoskeletal problems was the physiotherapist, by whom 52% of the women and 39% of the men had been examined or treated. Furthermore, 30% of the women and 31% of the men reported having been examined or treated by their general practitioner, 22% of the women and 16% of the men had been examined or treated by a specialist doctor, and 20% of the women and 26% of the men had consulted a chiropractor. In addition, 33% of the women and 15% of the men reported having consulted other kinds of treatment providers of conventional or alternative medicine. The most frequently reported reasons for being examined or treated for musculoskeletal problems were symptoms in the neck, upper and lower back.

According to Table 5 females had higher prevalence odds ratios for pausing from practising alone, taking sick-leave, and using painkillers than males. There was no significant difference between the genders when it came to pausing from rehearsals or omitting playing at concert, but the odds ratios and 95% confidence intervals shows a tendency towards it. Looking at the prevalence odds ratios for the instrument groups it showed that the woodwind section and the brass players, together named wind instruments, significantly paused less from planned playing activity no matter if it was practising alone, rehearsals, or concerts. Furthermore the woodwind players had a significantly lower odds ratio for sick-leave. The odds ratios for the instrument groups are listed in Table 6.

### Comparison with the Danish Workforce

The comparison of musculoskeletal problems in the symphony orchestra musicians with those in the general workforce in Denmark is illustrated in Figures 1 and 2. Six anatomic regions were compared: Regarding the neck, left shoulder, and left hand and wrist, significantly more musicians experienced symptoms within the last week as well as within the last year, and significantly more musicians had symptoms for more than 7 days and also for more than 30 days within the last year. For both genders, the left shoulder and left hand and wrist also accounted for the largest prevalence ratios of all compared regions.

Musicians of both genders had significantly more symptoms in the right shoulder within the last week and within the last year, and more had symptoms for at least 7 days, than seen in the general workforce. Significantly more female musicians also had right shoulder symptoms for more than 30 days. Regarding the right hand, significantly more female musicians than other female workers had symptoms within the last week and within the last year or had symptoms for at least 7 days. Also the male musicians had significantly more right hand and wrist symptoms within the last week than men in the general workforce, and significantly more male musicians than men in the workforce sample had right hand symptoms for more than 30 days within a year.

Lower back problems were less overrepresented in the musicians and were for both genders only significantly higher within the last seven days.

## Discussion

### Main findings

The main findings of this study were the very high prevalence of musculoskeletal symptoms in the neck, back, and upper extremities among professional symphony orchestra musicians and the considerable impact the symptoms had on the musicians' level of function in work with changed or impaired way of playing as well as outside work in both domestic and leisure time activities, and in sleep.

There was barely any statistically significant risk difference between the instrument groups, except that the woodwind players turned out to have a significantly lower risk for experiencing musculoskeletal problems and a significantly lower risk for consequences - such as impact on level of function - from musculoskeletal symptoms. In contrast there was a significant gender difference: Compared to the men, the females had a significantly higher risk for having symptoms, having the symptoms for more days, and females were also more affected by the consequences.

A comparison of the results for the musicians to the results for the general workforce showed that symptoms were significantly more frequent in more anatomic regions in musicians than in the general workforce.

### Interpretation of findings

There was a pronounced gender difference with female musicians having a higher prevalence of complaints than males. A similar gender difference was found in the general workforce. However, compared to the general workforce both genders of symphony orchestra musicians had a higher prevalence of musculoskeletal complaints; not only more symptoms but also symptoms lasting for more days, see Figure 1 and 2. For both genders, the left shoulder and left hand and wrist accounted for the largest prevalence ratios compared to the general workforce. These findings are consistent with the symphony orchestra musicians' monotonous working postures, which depending on the played instrument typically also comprise a prolonged static and/or repetitive dynamic but monotonous use of the left upper extremity. In general, musicians are furthermore often subject to prolonged static use of the neck and shoulder(s), monotonous repetitive use of joints in the upper limb(s), asymmetric body postures, or even a combination of these. A causal relationship between such ergonomic exposures and disorders in neck and shoulders is known [28]. Other studies have pointed out different ergonomic risks in string playing musicians and wind instrumentalists [15,29]. All in all it tends to indicate that the musicians' musculoskeletal symptoms are work-related, wholly or partially.

The musicians reported changed or impaired way of playing, difficulties in daily activities at home, in leisure time activities and in sleep as common consequences of musculoskeletal symptoms. That significantly more women experienced impaired playing, difficulties in daily activities at home and in sleep than men might be caused by biological gender differences such as strength, size, hormonal status, or be an expression of culturally different roles between the genders, e.g. women in Denmark taking a greater share of the housework than men [30-32]. Whatever the cause, female musicians generally had a higher risk for perceived symptoms, more days of symptoms, and for consequences due to the symptoms than male musicians had.

Between the instrument groups there was a remarkably little difference in risk for perceived symptoms as well as for experiencing consequences due to the symptoms. Especially when looking at the self-perceived symptoms, the woodwind players were the only group that stood out. The woodwinds had, however, a lower risk for musculoskeletal symptoms in different anatomic regions and a lower risk for consequences as well. The most remarkable might not be the woodwinds showing a lower risk for perceived musculoskeletal symptoms. More remarkable might be the fact that the majority of instrumentalists seemed so homogenous a group that they - compared to the high string players - barely showed any significant difference in symptoms risk amongst the instrument groups.

Taking into account the many analyses made on instrument group level, see Table 3, 4, and 6, and that results are stated with 95% confidence, we should not be blind to the risk of randomly false significant or insignificant results. The woodwinds' significantly different risk was consistent throughout most results, whereas the few significant findings for the other instrument groups appear more sporadically and should therefore be interpreted with more caution. But with this in mind, there was a tendency of low string players being at a higher risk for having neck and upper back symptoms for more days than the other instrument groups. Likewise a tendency was revealed indicating that brass players had a lower risk for pausing from practising and performing due to musculoskeletal symptoms.

To understand the musician as a patient it is important to look at the behavioural consequences as different ways of coping with musculoskeletal symptoms or even physically impairment or loss of function. This research demonstrated, that due to musculoskeletal symptoms significantly more musicians of both genders reported having paused from practicing at home/alone than having paused from rehearsals, cancelled playing at concerts or taking sick-leave. This behaviour pattern is consistent with the cultural discipline inherent in the occupation, which is based on a deep appreciation of the necessity for each instrument to contribute to a musical piece, and a pronounced understanding of being an integral member of one orchestra when performing, leading to a natural hierarchical order of importance of playing situations, with concert performances and orchestral rehearsals being the most important. A less idealistic reason would be that the musicians due to the competitive work environment felt forced to play despite there symptoms motivated by a concern for reprisal or dismissal. So despite their symptoms, the professional musicians will therefore be inclined to perform at rehearsals and concerts, and play less when practicing alone, where reducing their effort can aid their recovery without compromising their colleagues. Consumption of analgesics, presumably to avoid, reduce or delay the sensation of symptoms and their consequences, is another behavioural strategy. A similar behaviour is seen in athletes with exercise-induced muscle injury [33,34]. The most used analgesic was paracetamol, a non-prescription medicine that can also be bought outside pharmacies. Despite many musicians reporting difficulty in sleeping due to musculoskeletal symptoms, the use of sedatives and hypnotics was low.

As most symphony orchestra musicians have played their main instrument since childhood starting out playing it as a leisure time activity that later became a way of life; as they compete in an environment occupied by audition winners; as they always play with the same physical exposure to the instrument and many eventually acquire musculoskeletal problems; and as they often play despite their symptoms, the musicians could be considered comparable to elite sportsmen. Their musculoskeletal symptoms have also been suggested comparable to the overuse injuries experienced among athletes indicating a very high degree of strain and injury [35,36]. But whereas elite sportsmen often retire in their thirties after practicing their sport for two or three decades, symphony orchestra musicians can play their main instrument for up to 60 years or more before retirement, the last 40 years as professional full-time musicians. Playing in a professional symphony orchestra must be considered an elite occupation with a high risk of acquiring musculoskeletal symptoms.

### Strengths and limitations

The cross-sectional study design was the chosen method for collecting data to reveal the extent of certain health and working conditions among professional musicians and to facilitate comparison of data with a follow-up investigation. Early interviews ensured that topics included in the questionnaire were of relevance to the musicians and not just of interest to the research group. Questions on musculoskeletal problems were mainly built on known questionnaires in order to have standardised results which could be compared to other groups [24,25,27]. As with all self-administered questionnaires, there can be information bias. In an attempt to lower recall bias, most questions focused on the last 12 months or less and had preprinted answer categories. Furthermore, all participants were guaranteed full anonymity, which is considered of importance when responding to health issues, especially as here in a competitive environment.

Respondents were representative of the study population with respect to gender, age, and instrument groups. Despite the very satisfactory response rate of 78%, a possible selection bias risk was an over-representation of symptoms among the respondents in respect to non-respondents. Another selection bias risk could be the healthy worker selection, as symphony orchestra musicians are a highly selected group [12].

Mastering the instrument with rhythmic timing and perfect sound is a prerequisite for winning a position in an orchestra. This is the result of playing the main instrument for many years, of often having a music conservatory education, and of playing 6 or 7 days a week in order to maintain the level. The selection of the fittest must take into account the musician's psychological, audiological and musculoskeletal health, because performance anxiety, hearing disorders or musculoskeletal problems could necessitate giving up the dream of a place in a professional symphony orchestra [37-39]. Thus, selection based on health is already done before consideration is given to employment in a professional symphony orchestra.

### Generalisability

The group of respondents in the current study was representative of the whole occupational group in Denmark regarding participation by gender and by instrument group, two variables known for all members of the occupational group. Symphony orchestras are very similar worldwide with approximately the same instrumentation, the same hierarchical organization in the instrument groups, and the same way in which the instruments are played. The results are therefore indicative of the level of musculoskeletal problems of this occupational group world-wide, including the likelihood of a higher occurrence of musculoskeletal symptoms amongst female musicians. That the population of symphony orchestra musicians in this study was characterized by fewer and younger women than men was not caused by a drop-out of women from the orchestras but reflects what has largely been a male dominated profession in Denmark until about two decades ago, and which it still is in many countries.

### Recommendations for future research

Comparable to professional athletes or even soldiers, professional symphony orchestra musicians are an elite occupational group with specific physical demands in their work including the necessity to practice for long periods to maintain a high level of performance. Recommendations for systematic research sequences to prevent injuries in sportsmen and soldiers could therefore be expected to be transferred to professional symphony orchestra musicians successfully [40-42]. Applying a similar research strategy to professional musicians, we recommend: a) collecting valid baseline data to determine the extent of the problem, b) identifying the aetiology and risk factors of, and mechanisms underpinning injuries, c) introducing a preventive intervention, d) assessing the effect of the intervention by a follow-up study with collected data that are comparable to the baseline data, e) implementing effective and cost-effective interventions, and f) following-up to evaluate if implemented interventions retain their effectiveness.

## Conclusions

Within the last year most symphony orchestra musicians had experienced musculoskeletal symptoms in the neck, back or upper extremities. The symptoms impacted on their level of function in and outside work and were reflected in their health behaviour: Impact on playing, sick-leave, difficulty in daily activities at home, in leisure time, or in sleep, as well as use of analgesics and use of health care providers were all common consequences of the musculoskeletal symptoms in the occupational group of symphony orchestra musicians.

Compared to the high strings players the woodwinds had a significantly lower risk, assessed as odds ratio, for perceiving musculoskeletal problems as well as for experiencing consequences due to the musculoskeletal problems. Finally the woodwind players also had a lower risk for pausing music playing activities as well as for taking sick-leave due to musculoskeletal problems. The group of low string players showed the tendency for having symptoms for more days. Brass players showed a statistically significant lower risk for pausing from practising, rehearsals, or not playing at concerts.

Compared to a sample of the general workforce, symphony orchestra musicians of both genders had a higher prevalence of musculoskeletal symptoms, and the musicians also had more days of symptoms. Compared to the general workforce sample the distribution and frequency of symptoms in the symphony orchestra musicians would be indicative of their symptoms being work-related. Professional symphony orchestra musicians are a highly selected group and should be considered an elite occupation with a high risk of acquiring musculoskeletal symptoms.

## Competing interests

The authors declare that they have no competing interests.

## Authors' contributions

Conception and design of the study: HMP, JB, JWH. Data collection: HMP. Data analysis: HMP, JB, NW. Interpretation of data: CM, HMP, JB, JWH, NW. Writing the article: HMP, NW. Design of figures: CM, JB, HMP. Revising the article critically: CM, HMP, JB, JWH, NW. Final approval of the version to be published: CM, HMP, JB, JWH, NW. Guarantor of the paper: HMP, NW.

## Pre-publication history

The pre-publication history for this paper can be accessed here:

http://www.biomedcentral.com/1471-2474/12/223/prepub
